# Supplementary material for: Identification and functional analysis of non-coding regulatory small RNA FenSr3 in Bacillus amyloliquefaciens LPB-18
Source: PeerJ. 2023 May 15;11:e15236. doi: 10.7717/peerj.15236 (PMC10194069; doi:10.7717/peerj.15236)
Supplement: Supplemental Information 4 [file peerj-11-15236-s004.zip › KO/CK-vs-T1_map/map04340.html]

KEGG PATHWAY: Hedgehog signaling pathway - Reference pathway


|  |  |
| --- | --- |
| **Hedgehog signaling pathway - Reference pathway** |  |

[
Pathway menu
| Organism menu
| Pathway entry
| Show description
| User data mapping
]

The Hedgehog (Hh) signaling pathway has numerous roles in the control of cell proliferation, tissue patterning, stem cell maintenance and development. The primary cilium is an important center for transduction of the Hedgehog signal in vertebrates. In Hh's absence, the Ptch receptor localizes to the cilium, where it inhibits Smo activation. Gli proteins are phosphorylated by PKA, CKI and GSK3B and partially degraded into truncated Gli repressor form (GliR) that suppresses Hh target gene transcription in the nucleus. In Hh's presence, Ptch disappears from the cilium, and activated Smo contributes to the translocation of the protein complex Gli, Sufu, Kif7 to ciliary tip, where Gli dissociates from the negative regulator Sufu. The production of Gli activator form (GliA) occurs and the increased nuclear accumulation of GliA results in activation transcription of Hh target genes.

|  |  |  |
| --- | --- | --- |
| Reference pathway | 184% 150% 122% 100% 82% 67% 55% | 图片下载 |
